# Design and methodology of SNAP-1: a Sprint National Anaesthesia Project to measure patient reported outcome after anaesthesia

**DOI:** 10.1186/s13741-015-0011-2

**Published:** 2015-04-17

**Authors:** Suneetha Ramani Moonesinghe, Eleanor Mary Kate Walker, Madeline Bell, the SNAP-1 investigator group

**Affiliations:** UCL/UCLH Surgical Outcomes Research Centre, University College Hospital NIHR Biomedical Research Centre, London, NW1 2BU UK; National Institute for Academic Anaesthesia’s Health Services Research Centre, Royal College of Anaesthetists, Churchill House 35 Red Lion Square, London, WC1R 4SG UK

**Keywords:** Patient-reported outcome, Patient satisfaction, Anesthesia awareness, Epidemiology, Cohort study

## Abstract

**Background:**

Patient satisfaction is an important metric of health-care quality. Accidental awareness under general anaesthesia (AAGA) is a serious complication of anaesthesia care which may go unrecognised in the immediate perioperative period but leads to long-term psychological harm for affected patients. The SNAP-1 study aimed to measure patient satisfaction with anaesthesia care and the incidence of AAGA, reported on direct questioning within 24 h of surgery, in a large multicentre cohort. A secondary aim of SNAP-1 was to test the effectiveness of a new network of Quality Audit and Research Coordinators in NHS anaesthetic departments, to achieve widespread study participation and high patient recruitment rates. This manuscript describes the study methodology.

**Methods:**

SNAP-1 was a prospective observational cohort study. The study protocol was approved by the National Research Ethics Service. All UK NHS hospitals with anaesthetic departments were invited to participate. Adult patients undergoing any type of non-obstetric surgery were recruited in participating hospitals on 13th and 14th May 2014. Demographic data were collected by anaesthetists providing perioperative care. Patients were then approached within 24 h of surgery to complete two questionnaires—the Bauer patient satisfaction questionnaire (to measure patient reported outcome) and the modified Brice questionnaire (to detect possible accidental awareness). Completion of postoperative questionnaires was taken as evidence of implied consent. Results were recorded on a standard patient case report form, and local investigators entered anonymised data into an electronic database for later analysis by the core research team.

**Results:**

Preliminary analyses indicate that over 15,000 patients were recruited across the UK, making SNAP-1 the largest NIHR portfolio-adopted study in anaesthesia to date. Both descriptive and analytic epidemiological analyses will be used to answer specific questions about the patient perception of anaesthesia care overall and in surgical sub-specialties and to determine the incidence of AAGA.

**Conclusions:**

The SNAP-1 study recruited a large number of UK hospitals and thousands of perioperative patients using newly established networks in the UK anaesthetic profession. The results will provide benchmarking information to aid interpretation of patient satisfaction data and also determine the incidence of AAGA reported on a single postoperative visit.

**Electronic supplementary material:**

The online version of this article (doi:10.1186/s13741-015-0011-2) contains supplementary material, which is available to authorized users.

## Background

Patient feedback metrics are viewed as important assessments of the quality of health care. Broadly, the patient’s viewpoint can be sought in three domains. First, they can provide feedback on interactions with health-care professionals and specifically communications skills: colloquially, this might be referred to as the “bedside manner” test; and in the UK, this is a mandatory assessment for clinical doctors as part of the revalidation process. Second, patients can provide feedback on their *experience* of health care; such questionnaires would usually seek the patient viewpoint on a diverse range of issues such as cleanliness of the care environment, efficiency of treatment and services and the courtesy and trustworthiness of staff. Finally, patients may provide their views on the efficacy of their treatment, using patient-reported outcome measures (PROMs). The National Health Service (NHS) has a mandated programme of patient-reported outcome measures for a number of surgical procedures which are aimed at alleviating symptoms and improving health-related quality of life, such as hip and knee joint replacement and varicose vein repairs.

In perioperative anaesthesia practice, the patient perspective on their care can be sought through the administration of patient satisfaction measures. Patient satisfaction has been previously described as a construct which comprises a cognitive evaluation and an emotional response to the care received [1]. It is important to use an appropriately designed questionnaire to measure patient satisfaction, so that reliable and valid results are obtained. A systematic review has recently been published which qualitatively assessed the psychometric development and validation of published patient satisfaction questionnaires relating to anaesthesia practice [2]. This study concluded that the Bauer questionnaire [3] was amongst the best for use in the perioperative setting, both in terms of the rigour with which it was developed and the acceptability of the questionnaire to patients.

While it is of clear importance to measure patient experience and satisfaction, in anaesthetic practice, an important adverse outcome which is not commonly searched for or reported in routine practice is accidental awareness under general anaesthesia (AAGA). Estimates of the incidence of AAGA vary from 1–2 per 1,000 patients in clinical trials, [4-6] to considerably lower (approximately 1 in 15,000 or 20,000 general anaesthetics) in observational studies and surveys [7-9]. As with patient satisfaction, of paramount importance in being able to estimate the incidence of AAGA is the type of questionnaire or interview administered to patients. Traditionally, the Brice questionnaire and protocol has been adopted to elicit reports of AAGA—a series of five open-ended questions which are administered to the patient three times within 30 days of surgery [10]. A modified Brice questionnaire, which used closed questions with multiple choice answers, was subsequently adopted for a large randomised control trial evaluating the efficacy of a protocoled use of Bi-spectral Index monitoring in the prevention of AAGA [6].

While most anaesthetists will be aware of the availability both of patient satisfaction questionnaires and Brice methods, clinical experience tells us that they are infrequently used in routine practice in the United Kingdom (UK). Reasons for this may include a lack of familiarity with validated measures and a lack of benchmarking data to aid interpretation of patient satisfaction data. Furthermore, there are only limited data evaluating the reported incidence of AAGA where patients have been approached using a Brice-style questionnaire in a real-world setting. Therefore, the first UK Sprint National Anaesthesia Project (SNAP-1) aimed to prospectively measure patient reported outcome after anaesthesia in a large multicentre cohort. This paper describes the methods and analysis plan for the SNAP-1 study.

## Methods

### Aims and objectives

The aims of SNAP-1 are to report the descriptive and analytic epidemiology of the delivery and patient-reported outcomes of anaesthesia in the UK. Objectives are as follows:

1. To describe conduct of perioperative anaesthesia in the UK, including personnel, technology support and pharmacological management and postoperative resource utilisation;
2. To describe the characteristics of the patient population undergoing surgery involving an anaesthetist;
3. To measure patient satisfaction after anaesthesia using a validated survey on a national scale;
4. To determine the relationship between individual patient predicted risk using validated scoring systems and patient-reported postoperative outcome
5. To record the reported incidence of AAGA in routine UK practice using a modified Brice questionnaire administered within 24 h of surgery;
6. To determine associations between patient characteristics, procedural factors and adverse patient-reported outcomes

In addition to these main aims and objectives, a secondary aim of SNAP-1 is to determine the effectiveness of the Quality Audit and Research Coordinator (QuARC) network, at achieving local engagement and coordination with a national research project. The QuARC network was established by the National Institute for Academic Anaesthesia’s Health Services Research Centre (NIAA HSRC); all UK anaesthetic departments were asked to nominate a consultant anaesthetist as the QuARC, with the aim of liaising between the clinical department and the NIAA HSRC in national survey, audit, quality improvement and research projects. A particular aim of SNAP-1 was also to engage trainee anaesthetists in research. Previous surveys have shown that many trainees are unable to access opportunities to participate in research, despite a desire to do so; [11,12] therefore, SNAP-1 was proposed as an opportunity to address this gap.

### Study design and ethics

SNAP-1 was a multicentre observational study. Ethics approval was granted by the UK National Research Ethics Service (West Midlands Committee; REC reference 14/WM/0043). The study was approved as a research study, with implied consent being provided by patients upon completion of the questionnaires.

### Recruitment, inclusion and exclusion criteria

All adult (≥18 years) patients in participating hospitals undergoing any type of surgery or procedure under anaesthesia (local, regional or general) or sedation administered by an anaesthetist were eligible for inclusion. The following patient groups were excluded:

- Age less than 18 years
- Patients unable to understand spoken or written English
- Obstetric patients
- Patients too unwell or confused to be able to complete the questionnaire
- Patients who refused consent

The recruitment dates for SNAP-1 were 13 and 14 May 2014. All eligible patients having procedures involving an anaesthetist in participating on those dates were given a participant information sheet (Additional file 1) before their procedure, explaining that they would be approached postoperatively to complete the study questionnaires. No specific consent form was required as the ethics approval stated that completion of the follow-up questionnaires could be taken as implied consent: this was explained to patients in the participant information sheet. The target recruitment rate for the study was 7,500 patients.

### Dataset

The dataset comprised three questionnaires: a demographic questionnaire (completed by the perioperative anaesthetist at the time of surgery; Additional file 2), the Bauer patient satisfaction questionnaire and a modified Brice questionnaire (MBQ) (Additional file 3). The MBQ was adapted from that used in the BAG-RECALL study [6]. Demographic data were collected on all patients in participating centres who met inclusion criteria on the study days. The variables within the demographic questionnaire included patient factors, personnel factors and process factors. The patient risk variables were based on simple previously validated preoperative predictors of objective adverse postoperative outcomes. Patients were subsequently approached to complete the follow-up questionnaires (see Additional file 3) either on the day of surgery before hospital discharge (for ambulatory surgery patients) or within 24 h of surgery (for those undergoing inpatient surgery). Both the date of the procedure and the date of survey completion were noted for each patient. Data were collected on paper case report forms (CRFs) and subsequently transcribed by local investigators into electronic CRFs via a secure web-based data entry portal. A unique identifier for each case was generated through the creation of each new eCRF.

### Data management

The paper CRFs include fields for patient identifiers (name, date of birth and hospital number). These CRFs are securely stored at each participating site in accordance with the principles of information governance and good clinical practice. The electronic CRFs did not contain fields for patient identifiable information: therefore, each local investigator was required to keep a recruitment log to enable later identification of patients if required. Thus, no patient identifiable data was transferred outside the local hospital environment either electronically or on paper.

### Post-study investigator questionnaire

In order to assess the impact of this study on engagement with research amongst UK anaesthetists, a post-study questionnaire was issued to all registered investigators (see Additional file 4).

### Study coordination and funding

Investigators were sought from every anaesthetic department in the UK which provided perioperative services to adult non-obstetric patients. Site and investigator recruitment was facilitated through the Quality Audit and Research Coordinator (QuARC) network. Recruitment of trainee investigators was particularly encouraged. SNAP-1 was also adopted onto the National Institute for Health Research clinical research portfolio, thereby enabling Clinical Research Network support to be provided to participating hospitals.

Study management was led by a core group of investigators based at the UCL/UCLH Surgical Outcomes Research Centre and the NIAA’s HSRC. The core group consisted of the Chief Investigator, a trainee lead investigator and a study administrator based at the NIAA HSRC. Study oversight was provided by the Executive Management Board of the NIAA’s HSRC. The study sponsor was the University College London Hospitals NHS Foundation Trust. The study was funded through a grant of £18,069 from the National Institute for Academic Anaesthesia and through salary support (for the Chief Investigator and trainee lead investigator) from the University College London Hospitals NHS Foundation Trust and its National Institute for Health Research Biomedical Research Centre.

### Analysis plan

All results will be reported in accordance with the “Strengthening the Reporting of Observational Studies in Epidemiology” (STROBE) statement [13]. The main focus of analysis is to describe the epidemiology of the practice of anaesthesia and patient-reported outcome after anaesthesia in the overall cohort and, where feasible, in surgical sub-specialty groups and for specific surgical procedures.

#### Study recruitment and missing data

Study recruitment rates and missing data will be reported. The total number of sites that participated (broken down by country) will be presented in addition to the total number of patients recruited. The number of eligible patients who did not complete the questionnaires and the reasons reported for this will be stated. Comparison will be made of the demographics of patients who did and did not complete follow-up questionnaires. A description will be provided of any missing data points.

#### Descriptive epidemiology

The information collected from the patient demographic and perioperative questionnaire will be presented. The number of cases per specialty and type of procedure will be stated. Point estimates and ranges will be given for patient descriptors (for example, age, ASA grade, comorbidities and body mass index) in addition to detail of the use of long-term analgesics or benzodiazepines. The characteristics of surgery will be described by urgency, severity and length of operation. The personnel (i.e. seniority of the anaesthetist(s)) involved will be described as well as the type and site of anaesthesia induction. Anaesthetic technique and postoperative destination will be described and summarised both overall and for surgical sub-specialty groups.

The variables for three risk prediction/adjustment tools were included in the dataset: the population-based ASA grade; [14] the Surgical Risk Scale; [15] and the Surgical Outcome Risk Tool [16]. The latter two of these systems have been validated for the prediction of 30-day mortality after surgery. The predicted mortality based on these scoring systems will be presented for the overall patient population and for surgical sub-specialty groups. Data will be presented describing patterns of admission to high dependency or intensive care facilities compared with the predicted risk of 30-day mortality according to these systems, in order to assess compliance with national recommendations on postoperative care [17,18].

#### Bauer patient satisfaction questionnaire

For each of questions 1–10 (on anaesthesia-related discomfort), counts and percentages will be reported for each of the three potential responses. For questions 11–15 (which ask about satisfaction with anaesthesia care), counts and percentages of each of the four potential responses will be reported. Further analysis of the all these responses within different categories using chi-square testing with Bonferroni’s correction to compare outcomes between categories, such as (for example):

- Ambulatory surgery—comparisons by specialty and operative procedure
- Inpatient surgery—comparisons by specialty and operative procedure
- Elective/expedited versus immediate/urgent

Within surgical categories, comparison will be made between the responses to the patient satisfaction questionnaires depending on whether the questionnaire was administered on the day of surgery or day 1 postoperatively.

Univariate analysis will be used to demonstrate associations between patient factors and adverse outcomes, defined as the worst category answer for each question. Logistic regression will be used to determine independent risk factors for the adverse outcomes. Analyses will be conducted for the overall population and for surgical and patient categories where the sample size allows. The decision on whether to conduct regression analyses in particular subgroups will be informed by consideration of the principles described by Vittinghoff and McCulloch, including consideration of whether continuous or binary variables are included, the number of events per outcome variables and the sample size itself [19].

#### Modified Brice questionnaire (MBQ)

Counts and percentages for the responses to each question will be presented. Comparison will be made between the patients’ perception of the anaesthetic technique (general versus sedation or regional) and the actual anaesthetic technique.

The responses to the MBQ will be analysed to identify cases which may potentially represent AAGA. Both the responses to the closed multiple choice questions and free-text responses will be analysed for this purpose. If a case is found where any of these responses would be consistent with a potential case of AAGA, the lead local investigator for the site where the patient was anaesthetised will be informed and asked to follow up the case according to their local departmental guidelines. Local investigators will be asked to provide structured feedback to the core research team. Following review of information received from local investigators, the case will be classified as either proven awareness, not awareness, or unable to determine.

As the SNAP-1 study only asks patients about awareness once within 24 h of surgery, the results will be extrapolated to determine an estimate of the likely actual incidence of awareness, based on the results of previous studies which used three separate interviews (on approximately day 0, days 1–2 and day 30 postoperatively) and reported the incidence of awareness occurring on each day [20]. Depending on the event rate and sample size, regression analyses to determine independent risk factors for awareness may be undertaken.

## Results and discussion

SNAP-1 follows similar methodology to previous studies of surgical [21] and anaesthesia-related epidemiology, [22] in adopting a “snapshot” approach to gather a large sample of data in a short time frame. However, these studies did not require patient consent or any interaction with patients outwith the usual practice of the anaesthesia or perioperative team. Thus, to our knowledge, this is the first such study in the UK to measure patient-reported outcome related to anaesthesia using this methodology. Preliminary analyses indicate that this is the highest recruiting NIHR portfolio-adopted consenting research study in anaesthesia to date.

SNAP-1 has introduced the concept of using a validated survey to measure patient satisfaction with anaesthesia to the majority of UK hospitals. It is hoped that individual departments will reflect on the resources that were required to facilitate the study and use this to inform future attempts to measure patient satisfaction for the purposes of quality improvement, research and revalidation. The post-study survey will assess the impact of the study on anaesthetists’ willingness to engage with the measurement of patient satisfaction and the screening of patients for AAGA in routine practice.

Once analysed and reported, it is hoped that SNAP-1 results will inform the development of patient information leaflets for different types of surgery, which can be used to better inform patients about the likely short-term outcomes of their anaesthetic and surgical experience. Summary national data should provide benchmarking information for future local and national surveys of patient-reported outcome after anaesthesia.

The study has received little primary funding and may provide important data on the feasibility of conducting such large-scale studies on a limited budget. While the study methodology was simple and the intervention (the administration of a questionnaire) was not complex, the logistics of coordinating a study which required individual Research and Development department approval in all four UK-devolved nations was considerable. The post-study investigator questionnaire should improve our understanding of the baseline level of experience of “non-academic” anaesthetists with research and research methodology and the potential benefits (and pitfalls) of engaging with clinical research for both trainees and career-grade doctors. Additionally, it will provide information about the motivation for anaesthetists’ participation in this study, including whether the promise of acknowledgment on study manuscripts was a significant incentive to take part. (see Additional file 5).

## Conclusions

SNAP-1 should provide new and important data on patient-reported outcome after anaesthesia, the incidence of AAGA in a “real-world” clinical setting and the impact of participation in nationally coordinated patient-focussed research for clinical anaesthetists in the UK, particularly trainees.

## Additional files

Additional file 1:
**Participant information sheet.**
Additional file 2:
**Patient demographics questionnaire.**
Additional file 3:
**Bauer and Brice questionnaires.**
Additional file 4:
**Post-study investigators questionnaires.**
Additional file 5:
**SNAP-1 investigator list (collaborators).**

## Abbreviations

AAGA: Accidental awareness under general anaesthesia
SNAP-1: Sprint national anaesthesia project
QuARC: Quality audit and research coordinator
MBQ: Modified brice questionnaire
BPS: Bauer patient satisfaction questionnaire
NIAA: National institute for academic anaesthesia
HSRC: Health services research centre
ASA: American society of anesthesiology physical status score
