# Supplementary material for: Design and methodology of SNAP-1: a Sprint National Anaesthesia Project to measure patient reported outcome after anaesthesia
Source: Perioper Med (Lond). 2015 Apr 17;4:4. doi: 10.1186/s13741-015-0011-2 (PMC4422533; doi:10.1186/s13741-015-0011-2)
Supplement: Additional file 1: — Participant information sheet. [file 13741_2015_11_MOESM1_ESM.doc]

**Patient Information Sheet:**

**Sprint National Anaesthesia Project (SNAP-1) Patient Survey on Quality of Anaesthesia in UK Hospitals.**

We are conducting a study in hospitals throughout the UK and would like to invite you to take part in it. Before you decide whether to take part or not you need to understand why the study is being done and what it would involve for you. Please take time to read the following information carefully & decide if you want to take part. Talk to others about the study if you wish. Ask us if there is anything that is not clear or if you would like more information.

**What is the purpose of the study?**

We want to find out what patients think of the anaesthetic care that they receive and whether you have suffered from any discomfort after your operation that could be related to the anaesthetic. By asking patients in hospitals throughout the UK, we hope to get a lot of information about whether certain types of operations or anaesthetics lead to high or low levels of patient satisfaction. Armed with this information, we would in the future hope to improve any areas where there were lower levels of patient satisfaction.

We also want to find out about the very rare, but potentially distressing, experience of awareness under anaesthesia. This is when a patient becomes conscious or partly conscious during an anaesthetic and can remember things that have happened during the operation. We are interested in finding out how often this occurs in the UK. At present we believe it happens to less than 1 in every 14,000 people.

**Why have I been invited?**

We are asking all patients, in hospitals throughout the UK who are having operations on Tuesday 13^th^ and Wednesday 14^th^ May 2014 to complete 2 short questionnaires.

**Do I have to take part?**

Taking part is entirely voluntary and it is up to you to decide whether you do so. We will describe the study and go through this information sheet, which we will then give to you. You are free to withdraw at any time without needing to give a reason and this would in no way affect the standard of care you receive.

**What will I have to do?**

After your operation you will be visited by a member of the anesthetic team, who will give you 2 short questionnaires (each 1 side of A4 paper) which should take no more than 5 minutes in total to complete. By completing the questionnaires you are giving permission for us to keep the information you provide and analyse it for our study.

**What are the possible disadvantages and risks of taking part?**

We don’t think there are any disadvantages or risks of taking part. Filling in the questionnaires should be straightforward as they mostly consist of tick boxes. Both questionnaires have been used before in other groups of patients with no reports of significant ill effects. There is a small possibility that answering the questionnaires may cause you to worry or feel anxious about your anaesthetic and if, having filled in either of the questionnaires, you have any questions or concerns there will be an opportunity to discuss these with the member of the anaesthetic team when they collect the forms.

**What are the possible benefits of taking part?**

We cannot promise the study will help you directly but we hope that the information we get from this study will help to improve the quality of anaesthetic care delivered by hospitals in the future. You will be able to get feedback on the findings of the study via the SNAP-1 website (see details below).

**What do I do if there is a problem or I wish to make a complaint?**

Every hospital trust in the country has a Patient Advice and Liaison Service (PALS) who can be contacted and will provide information about the NHS complaints procedure, including how to get independent help if you want to make a complaint.

**Will my taking part in the study be kept confidential?**

Yes. We will follow ethical and legal practice and all information about you will be handled in confidence. We will do everything we can to protect your privacy.

**What will happen to the answers that I provide in the questionnaires?**

The local study leader will transfer your answers from the paper questionnaires via a secure web-based data collection tool onto a computer database. None of your personal details will be transferred out of the local hospital either electronically or on paper. The anonymised responses from hospitals across the U.K. will be analysed by a team of researchers based at University College Hospital in London.

**What will happen to the results of the study?**

The results from the study will be published on the SNAP-1 website by early 2015. We also intend to produce both oral and written reports of the results but no references will be made that could link you personally to the study.

**Who is organising and funding the study?**

The study is being organised & funded by the National Institute of Academic Anaesthesia Health Services Research Centre (HSRC) & the Royal College of Anaesthetists.

**Who has reviewed the study?**

All research in the NHS is looked at by independent group of people known as a Research Ethics Committee whose job is to protect your safety, rights, wellbeing and dignity. This study has been reviewed and approved by the East Midlands Research Ethics Committee on behalf of the Health Research Authority.

**Further information and contact details**

Website: http://www.niaa.org.uk/SNAPs

Study email address: snap1@rcoa.ac.uk

Local lead name and contact details:

Local PALS contact details:
