# Supplementary material for: Design and methodology of SNAP-1: a Sprint National Anaesthesia Project to measure patient reported outcome after anaesthesia
Source: Perioper Med (Lond). 2015 Apr 17;4:4. doi: 10.1186/s13741-015-0011-2 (PMC4422533; doi:10.1186/s13741-015-0011-2)
Supplement: Additional file 2: — Patient demographics questionnaire. [file 13741_2015_11_MOESM2_ESM.docx]

**SNAP-1: Patient demographics (to be completed at time of surgery)**

*See below for definitions for questions marked **

**Patient name:**

**Hospital number: DOB: Age:**

**SNAP-1 unique identifier (generated once uploaded to web tool): ___________________________________**

**Date of operation:** 13/05/14 ☐ 14/05/14 ☐ **Sex:** Male ☐ Female ☐

**Operation name: __________________________________________________________________________________**

**ASA grade:** 1☐ 2 ☐ 3 ☐ 4 ☐ 5 ☐

**Surgical urgency:*** Elective ☐ Expedited ☐ Urgent ☐ Immediate ☐

**Surgical severity:*** Minor ☐ Intermediate ☐ Major ☐ Complex ☐

**Comorbidities:**

Congestive Cardiac Failure:* Yes ☐ No ☐

Previous stroke / TIA: Yes ☐ No ☐

Cancer within past 5 years: Yes ☐ No ☐

Obesity (BMI ≥ 30): Yes ☐ No ☐

**Is the patient on long-term analgesics or benzodiazepines?**

Opiates/Opioids: Yes ☐ No ☐ Benzodiazepines: Yes ☐ No ☐

NSAIDs / COX inhibitors: Yes ☐ No ☐ Neuropathic pain meds: Yes ☐ No ☐

---------------------------------------------------------------------------------------------------------------------------------------**Congestive cardiac failure:** history of congestive heart failure, pulmonary oedema, or paroxysmal nocturnal dyspnoea; physical examination showing bilateral rales or S3 gallop; or CXR showing pulmonary vascular redistribution

**Surgical severity:**

**Minor:** e.g. carpal tunnel, MUA, arthroscopy: procedure generally lasts <30min

**Intermediate:** e.g. hernias, ACL reconstruction, varicose veins: procedure generally lasts <1 hour

**Major:** e.g. lap cholecystectomy, primary joint replacement

**Complex:** e.g. open cavity surgery, major laparoscopic surgery, organ resections, bilateral or revision joint replacements

**Surgical urgency:**

**Immediate**: immediate life, limb or organ-saving intervention. Normally within minutes of decision to operate.

**Urgent**: surgery for acute onset / clinical deterioration of potentially life-threatening conditions that may threaten survival of limb or organ; fixation of many fractures & for relief of pain / distressing symptoms. Normally within hours of decision to operate.

**Expedited**: early treatment for a condition not immediately threatening to life /limb /organ survival. Normally within days of decision to operate.

**Elective**: intervention booked in advance of routine admission to hospital. Timing to suit patient, hospital & staff.

**Perioperative care:**

**Anaesthetic induced by** (please tick all that apply)**:**

Consultant ☐ Trainee/Trust grade junior ☐ Non-consultant grade senior ☐ PA(A) ☐

**Induction of GA:** Inhalational ☐ Intravenous ☐ Not GA ☐

**Where did induction take place?** Anaesthetic room ☐ Operating theatre ☐

**Maintenance intraoperative anaesthesia** (please tick all that apply)**:**

GA ☐ Inhalational anaesthetic ☐ TIVA ☐

Epidural ☐ Spinal ☐ Combined spinal & epidural ☐

Surgical infiltration with LA ☐ Nerve block ☐

Awake/light sedation (response to speech) ☐

Deep sedation (no response to speech, response to physical stimulus) ☐

**Neuromuscular blocking agent used?** Yes ☐ No ☐

**Induction and intraoperative analgesia administered** (please tick all that apply):

Alfentanil ☐ Fentanyl ☐ Morphine ☐ Remifentanil ☐

Paracetamol ☐ NSAID ☐ Ketamine ☐ Clonidine ☐

Other (please specify):

**Antiemetic administered** (please tick all that apply):

5-HT_3_ antagonist ☐ Dexamethasone ☐ Cyclizine ☐ Prochlorperazine ☐ Droperidol ☐

Other (please specify):

**Monitoring used:**

End tidal anaesthetic agent concentration: Yes ☐ No ☐ Not applicable ☐

Bispectral Index (BIS)/E-Entropy/Narcotrend used: Yes ☐ No ☐ Not applicable ☐

**Duration of surgery (not including anaesthesia time):**

<30min ☐ 30m-1h ☐ 1-2h ☐ >2h ☐

**Postoperative destination/ level of care:**

Home (day case procedure) ☐ Inpatient ward ☐

High Dependency Unit (Level 2) ☐ Intensive Care Unit (Level 3) ☐

*Many thanks for taking the time to complete this information.*
