# Supplementary material for: Design and methodology of SNAP-1: a Sprint National Anaesthesia Project to measure patient reported outcome after anaesthesia
Source: Perioper Med (Lond). 2015 Apr 17;4:4. doi: 10.1186/s13741-015-0011-2 (PMC4422533; doi:10.1186/s13741-015-0011-2)
Supplement: Additional file 3: — Bauer and Brice questionnaires. [file 13741_2015_11_MOESM3_ESM.docx]

**Today’s date:**

**At any stage after your operation have you had the following (please tick one box for each question 1-10):**

**No Yes, Yes,**

**moderate severe**

1. Drowsiness ☐ ☐ ☐
2. Pain at the site of surgery ☐ ☐ ☐
3. Thirst ☐ ☐ ☐
4. Hoarseness ☐ ☐ ☐
5. Sore throat ☐ ☐ ☐
6. Nausea or vomiting ☐ ☐ ☐
7. Feeling cold ☐ ☐ ☐
8. Confusion or disorientation ☐ ☐ ☐
9. Pain at the site of the anaesthetic injection ☐ ☐ ☐
10. Shivering ☐ ☐ ☐

**Satisfaction with anaesthesia care (please tick one box for each question 11-16):**

1. How satisfied were you with the information you were given by the anaesthesist before the operation?

☐ ☐ ☐ ☐

**Very satisfied Satisfied Dissatisfied Very dissatisfied**

1. How satisfied were you waking up from anaesthesia?

☐ ☐ ☐ ☐

**Very satisfied Satisfied Dissatisfied Very dissatisfied**

1. How satisfied have you been with pain therapy after surgery?

☐ ☐ ☐ ☐

**Very satisfied Satisfied Dissatisfied Very dissatisfied**

1. How satisfied were you with treatment of nausea and vomiting after the operation?

☐ ☐ ☐ ☐

**Very satisfied Satisfied Dissatisfied Very dissatisfied**

1. How satisfied were you with the care provided by the department of anaesthesia in general?

☐ ☐ ☐ ☐

**Very satisfied Satisfied Dissatisfied Very dissatisfied**

1. Would you recommend this anaesthetic service to friends and family? **YES☐**  **NO☐**

**17. Were you expecting to have a general anaesthetic (be completely asleep) for this operation?** No ☐ Yes ☐

**18. What is the last thing you remember before going to sleep (please tick one box)?**

-Being in the pre-operative area ☐ -Seeing the operating room ☐

-Being with family ☐ -Hearing voices ☐

-Feeling mask on face ☐ -Smell of gas ☐

-Burning or stinging in the IV line ☐ -Not applicable ☐

-Other [Please write below]:

**________________________________________________________________________________________________________________**

**19. What is the first thing you remember after waking up (please tick one box)?**

-Hearing voices ☐ -Feeling breathing tube ☐

-Feeling mask on face ☐ -Feeling pain ☐

-Seeing the operating room ☐ -Being in the recovery room ☐

-Being with family ☐ -Being in the intensive care unit ☐

-Nothing ☐ -Not applicable ☐

-Other [Please write below]:

**________________________________________________________________________________________________________________**

**20. Do you remember anything between going to sleep and waking up (please tick box)?**

**-**No ☐

-Yes: -Hearing voices ☐ -Hearing events of the surgery ☐

-Unable to move or breathe ☐ -Anxiety/stress ☐

-Feeling pain ☐ -Sensation of breathing tube ☐

-Feeling surgery without pain ☐ -Not applicable ☐

-Other [Please write below]

**________________________________________________________________________________________________________________**

**21. Did you dream during your procedure (please tick box)?**

-No ☐ -Yes ☐

-What about [Please write below]:

**________________________________________________________________________________________________________________**

**22. Were your dreams disturbing to you (please tick box)?**

-No ☐ -Yes  **☐**

**23. What was the worst thing about your operation (please tick box)?**

-Anxiety ☐ -Pain ☐

-Recovery process ☐ -Unable to carry out usual activities ☐

-Awareness ☐ -Other [Please write below]:

**Thank you for taking the time to complete this questionnaire!**
