# Supplementary material for: Design and methodology of SNAP-1: a Sprint National Anaesthesia Project to measure patient reported outcome after anaesthesia
Source: Perioper Med (Lond). 2015 Apr 17;4:4. doi: 10.1186/s13741-015-0011-2 (PMC4422533; doi:10.1186/s13741-015-0011-2)
Supplement: Additional file 4: — Post-study investigators questionnaires. [file 13741_2015_11_MOESM4_ESM.docx]

**Post study questionnaire for Local Lead Investigators**

1. **Are you the QuARC (Quality Audit and Research Coordinator) for your department?**

- Yes / No / Not sure

1. **Please name the region in which you work:**

- List of regional training areas

1. **Do you consider your anaesthetic department to be research active?**

- Yes / No / Not sure / Freetext option

1. **Before SNAP-1, did you consider yourself to be research-active within your department?**

- Yes / No / freetext option

1. **What encouraged you to take part in SNAP-1? (please tick all that apply)**

- I thought the study was interesting / worthwhile
- I was told / asked to take part by my department
- The study was led by the National Institute for Academic Anaesthesia Health Services Research Centre
- The study was linked to the NAP5 project
- The promise of being recognized for my work by being a named collaborator in SNAP-1 reports / publications
- The study was on the NIHR portfolio
- Other (freetext option)

1. **Were there any negatives about taking part? (free text for ‘yes’)**
2. **What specific role(s) did you play in SNAP-1 (please tick all that apply)**

- Coordinating your department’s study participation
- Liaising with your R and D department
- Liaising with central SNAP-1 coordination
- Administering patient information sheets
- Administering postoperative questionnaires
- Answering patient queries
- Entering data into database
- Other (please state)

FREETEXT OPTION

1. **How would you rate your experience of liaising with your local R&D department?**

- Poor / satisfactory / good / did not knowingly have anything to do with them

Please provide further information if you wish (freetext)

1. **To obtain permission to take part in SNAP-1, was it clear who to contact within your R&D department?**

- Yes, already understood process
- yes, easy to follow the local pathway to gain permission
- No, not particularly easy to understand process
- I had no contact with my R&D department

**10. Have you been an investigator on a research study before?**

- Yes / No

**11. Were you aware of Good Clinical Practice (GCP) before taking part in SNAP-1?**

- Yes / No

**12. Had you completed GCP training within the past two years prior to your involvement in SNAP-1?**

- Yes / no

**13. Have you completed GCP training as a result of your involvement in SNAP-1?**

- Yes / no / not applicable because I already had it

**14. Would you be prepared to be a Local Lead Investigator (hospital / Trust lead) in future SNAP research projects?**

- Yes / No / Maybe (Freetext option)

**15. Before taking part in SNAP-1, did you have any experience of measuring patient satisfaction in relation to your own anaesthetic practice?**

Yes / No / Not sure / Not applicable

**16. Would you now consider using a patient satisfaction survey such as that used in SNAP-1 to measure patient reported outcome for your own practice (for the purpose of quality improvement, audit and/or revalidation). Please provide further information below if you would like to**

Yes / No / Not sure / Not applicable

FREETEXT OPTION

**17. Before SNAP-1, did you routinely consent patients for the risk of accidental awareness under general anaesthesia?**

Yes / No / Depends on the patient or procedure / Not applicable

**18. Would you now consider using the Brice questionnaire to evaluate whether the patient had experienced accidental awareness under general anaesthesia**

Yes / No / Depends on the patient or procedure / Not applicable

**19. What would you do differently for future SNAP projects?**

FREETEXT

**20. Please use this space to provide us with any other feedback**

FREETEXT

Thank you for taking part in this survey

**Post-study questionnaire for local investigators**

1. **What is your job title?**

- Medical Student
- Foundation year doctor
- Trainee (ST1-7)
- Trust grade doctor (ST1 – 7 equivalent)
- Associate Specialist / Senior Staff grade
- Consultant
- Research nurse
- Data manager
- Other (please state)

1. **Please name the region in which you work:**

- List of regional training areas

1. **Do you consider your anaesthetic department to be research active?**

- Yes / No / Not sure / Freetext option

1. **Before SNAP-1, did you consider yourself to be research-active?**

Yes / No / freetext option

1. **What motivated you to take part in SNAP-1? (please tick all that apply)**

- I thought the study was interesting / worthwhile
- I was told / asked to by my department
- The study was led by the National Institute for Academic Anaesthesia Health Services Research Centre
- The promise of being recognized for my work by being a named collaborator in SNAP-1 reports / publications
- The study was on the NIHR portfolio
- Other (freetext option)

1. **Were there any negatives about taking part? (free text for yes)**
2. **What specific role(s) did you play in SNAP-1 (please tick all that apply)**

- Coordinating your department’s study participation
- Liaising with central SNAP-1 coordination
- Administering patient information sheets
- Administering postoperative questionnaires
- Answering patient queries
- Entering data into database
- Other (please state)

FREETEXT OPTION

**8. Have you been an investigator on a research study before?**

- Yes / No

**9. Were you aware of Good Clinical Practice (GCP) before taking part in SNAP-1?**

- Yes / No

**10. Had you completed GCP training within the past two years prior to your involvement in SNAP-1?**

- Yes / no

**11. Have you completed GCP training as a result of your involvement in SNAP-1?**

- Yes / no / not applicable because I already had it

**12. Before taking part in SNAP-1, did you have any experience of measuring patient satisfaction in relation to your own anaesthesia practice?**

Yes / No / Not sure / Not applicable

**13. Would you now consider using a patient satisfaction survey such as that used in SNAP-1 to measure patient reported outcome for your own practice (for the purpose of quality improvement, audit and/or revalidation). Please provide further information below if you would like to**

Yes / No / Not sure / Not applicable

FREETEXT OPTION

**14. Before SNAP-1, did you routinely consent patients for the risk of accidental awareness under general anaesthesia?**

Yes / No / Depends on the patient or procedure / Not applicable

**15. Would you now consider using the Brice questionnaire to evaluate whether the patient had experienced accidental awareness under general anaesthesia**

Yes / No / Depends on the patient or procedure / Not applicable

**16. What would you do differently for future SNAP projects?**

**17.Please use this space to provide us with any other feedback**

- FREETEXT

Thanks for taking part in this survey
