# Supplementary material for: Design and methodology of SNAP-1: a Sprint National Anaesthesia Project to measure patient reported outcome after anaesthesia
Source: Perioper Med (Lond). 2015 Apr 17;4:4. doi: 10.1186/s13741-015-0011-2 (PMC4422533; doi:10.1186/s13741-015-0011-2)
Supplement: Additional file 5: — SNAP-1 investigator list (collaborators). [file 13741_2015_11_MOESM5_ESM.docx]

**Central SNAP-1 Organisation**

Chief Investigator

S Ramani Moonesinghe

Trainee Lead Investigator

Eleanor MK Walker

Study Coordinator

Madeline R Bell

National Institute of Academic Anaesthesia

Sharon Drake

Mary Casserly

Dimitri Papadimitriou

Jose Lourtie

Martin Leuwer

National Institute of Academic Anaesthesia’s Health Services Research Centre

Michael PW Grocott

Jonathan Benn

Timothy M Cook

Dave Murray

Charles Matthew Oliver

Rupert M Pearse

David Saunders

Annique Simpson

UCL/UCLH Surgical Outcomes Research Centre

Oliver Boney

Sarah Barnett

Michael Galsworthy

Michael G Mythen

Net Solving Ltd

Martin Cripps

Paul Cripps

Rayzume Ltd

Jon Fuller

Research and Audit Federation for Trainees

Thomas Clark ^g^

Benjamin Harris ^e^

Charlotte Small ^I^

Jason Lie^c^

**National Study Groups**

* indicates Local Lead Investigator for a Trust / Health Board

Trainee Research Networks

^a^ indicates membership of AARMY

(Anaesthetic Audit and Research Matrix of Yorkshire)

^b^ indicates membership of INCARN.NET

(Intensive Care and Anaesthesia Research Network North East Trainees)

^c^ indicates membership of NWRAG

(North-West Research and Audit Group)

^d^ indicates membership of SHARC

(South Yorkshire Hospitals Audit and Research Collaborative)

^e^ indicates membership of SPARC

(South coast Perioperative Audit and Research Collaborative)

^f^ indicates membership of STAR

(Severn Trainees Anaesthetic Research group)

^g^ indicates membership of SWARM

(South West Anaesthesia Research Matrix)

^h^ indicates membership of WAAREN

(Welsh Anaesthesia Audit and Research Engagement Network)

^I^ indicates membership of WM TRAIN

(West Midlands Trainee Research in Anaesthesia and Intensive care Network)

**Northern Ireland**

Belfast Health and Social Care Trust

Mona Behravesh

Joanne Colgan*

Eva Corcoran

Samuel Dawson

Andrew Eggleton

Grainne Fitzpatrick

Denver Glasgow

Mary Clare Kelly*

Barbara MacAfee

Killian McCourt*

Claire Montgomery

Alexandra Murphy

Gareth Paul

James Quinn

Lloyd Turbitt

Alison White

Callum Wilson*

Northern Health and Social Care Trust

David Johnston

Kumar Manmadha Kada-Venkata*

Helen Lindsay

Geoffrey Wright*

Western Health and Social Care Trust

Vinanti Cherian*

William Holmes

Nauman Iftikhar

Eireann Kerr

Muhammad Usman Latif*

Madalina McCrea

Karen Orr

Paula Claire Pyper

South Eastern Health and Social Care Trust

Paul Foley*

Lori Lindsay

Simon Marcus

Helen Murray

Nicola Wallace

Southern Health and Social Care Trust

Patricia Anagnostides

Rebecca Barr

Gail Browne*

Lisa Clarke

Rachel Copeland

**Scotland**

NHS Ayrshire & Arran

Andrew Clark

Susan Livingstone

Michael McLaughlin

Joellene Mitchell*

Peter O'Brien*

Ker Wei Tan

NHS Dumfries & Galloway

Alexander Arthur

Naomi Cassells

Vivien Edwards*

Benjamin Vowles

NHS Fife

Linsey Burd

Simon Chillingworth

Sarah Fadden

Rachel Gill

Katie Hunter*

Savini Wijesingha

NHS Forth Valley

Deirdre Conway

Stephen Hickey

Yuvaraj Kummur

Srikanth Lakshminarayan

Reza Noori

Monojit Paul

Azfer Usmani*

NHS Grampian

Adriana Botello

Jill Davidson

Bianca Ebtehadj

Callum Kaye

Naveen Kirodian

R Nanda Kumar

Andrew Laurie

Amr Mahdy*

Aimee Meechan

Bahadur Niazi

Louise Peet

NHS Greater Glasgow & Clyde

Ravi Agaram

Rajib Ahmed

Thomas Ballantyne

Julie Campbell

Anna Cormack

Richard Cowan

Barbara A. Crooks*

Rachel Darling

John Dolan

Nicola Doody

Rachel Fulton

Michael Gardner

Adam Glass

Stuart Hannah

Roderick Hamilton*

Johann Harten*

Sue Hutton

Rebecca Jadhav

Kenneth James

Kerry Litchfield

Adam Livingston

Katherine Livingstone

Eilidh MacDonald

Naomi May

Myra McAdam

Elizabeth McGrady

Rhys Millington

Dalia Mitra

Jane Morrison

Helen Rhodes

Emma Rhodes

Drew Smith

Manfred Staber*

Vanessa Vallance

Daphne Varveris*

Douglas Walker*

Louise Welsh

Simon Young

NHS Highland

Jayne Halcrow

Jacqueline Howes*

Emily Robertson

David Robinson*

NHS Lanarkshire

Ali Atrah

Roddy Chapman*

Jonathan Edgar*

Juan Escuder*

Sumit Gajree

Keith Hodgson

Chris Lochrin

Christopher Love

Naveeta Maini

Mark Mifsud

Ayman Mustafa

Khaled Razouk*

Jose Soriano

Dominic Strachan

Tamas Szelei

Ruth Taggart

NHS Lothian

R Peter Alston*

Mohammad Zeshan Bhatti

Ellie Cox

Vanessa Humphrey

Faisal Jafar

Keith Kelly

Evangelia Matiaki

Stuart McLellan

John McLenachan

Laura McRae

Martin Slattery

Catriona Williamson

NHS Orkney

Paul Cooper*

NHS Shetland

Catriona Barr*

Keh Wei Kong

NHS Tayside

Elizabeth Broadbent

Natalie Clark

May Mok*

Shirley Moore

NWTCB Golden Jubilee National Hospital

Stefan Schraag*

Firas Abu-Eishe

Bhushan Joshi

Isma Quasim

**Wales**

Abertawe Bro Morgannwg University Health Board

Paul Carter

Benjamin O Donovan

Benjamin Eagle

James Farrant^h^

Maryna Garmash

Jishar Abdul Kader

Bethan Morris

Shilpa Rawat*

Robin Spacie

Ramakrishna Suresha

Gareth Taylor

Thomas White

Viju Varadarajan*

Aneurin Bevan Health Board

Timothy Alce

Lowri Bowen

Kevin Draper^h^

Rebecca Jackson

Karen Meacher

Samuel Thomas Meinrad Sheppard

Thomas Morgan-Jones*

James Tozer*

Betsi Cadwaladr University Health Board

Justine Angrave

Craig Beaton^h^

Gillian Bennett

Stephan Clements*

Naomi Daws

Chris Goodman

Bethan Griffiths

Elizabeth Jones

Stanley Jose

Nia Kelly

Piotr Kucharski

Chris Littler*

Muthuraja Marimuthu

Doug Morgan

Richard Pugh*

Keivn Rafferty

Nirojan Sivapathasundararajah

Cardiff and Vale University Health Board

Ceri Beynon

Poonam Bopanna

Clare Dallimore

Hywel Evans

Naomi Goodwin*

Laura Jackson^h^

Kathryn Lloyd-Thomas

Zarah Paris

Anthony Short

Navneet Sinha

Mari Thomas

Cwm Taf Health Board

Megan Burton

Louise Elliott

Najia Hasan^h^

Valerie Hilton*

Kathryn James

Jessica Laura Lowe

Anna Musgrave^h^

Omar Alex Pemberton*

Katie Ross

Rosie Scott

Hywel Dda Health Board

Sunita Agarwal*

Brian Campbell*

Stuart Gill*

Peter Havalda

Rhiannon Lewis

Christopher Nwaefulu

Vanie Rajesh

Abdulaziz Refaet

Shan Sekaran

Gopinath Selvaraj

Norbert Skarbit

Alun Thomas

Edward Todd

Adam Wood

**England**

Aintree University Hospitals NHS Foundation Trust

Liz Clark

Sarah El Sheikha

Sam Howitt

Andrew Langdon

Simon Mercer

Dermot Moloney*

Mari Roberts

Ravish Shetty

Emma Welfare

Airedale NHS Foundation Trust

Shivanandaswamy Kashimutt

Wendy Lum Hee*

Claire Kurasz

Paul Underwood ^a^

Ashford and St Peter's Hospitals NHS Foundation Trust

Melinda Brazier

Sam De Silva

Samson Ma

Louise McDevitt

Katharine O'Rourke

Tasmin Patel

David Robinson*

Vatshalan Santhirapala

Oliver Sykes

Yize Wan

Barking, Havering and Redbridge University Hospitals NHS Trust

Ritesh Ganesh

Mahir Khalil

Hannah Lewis

Shabir Qadri*

Nadeem Shakir

Sheelaj Sharma

Barnet and Chase Farm NHS Hospitals Trust

Kakali Chattopadhyay

Nicolas Hooker

Rajeev Jha*

Janine Mawby

James Parry

Raj Shah

Evan Wild

James Thomas

Barnsley Hospital NHS Foundation Trust

Oliver Ashby

Sunil Kumar Chaurasia*^d^

Rory Colhoun ^d^

Elinor Cromarty ^d^

Dawn Johnson

Wei Yang Low ^d^

Barts and The London NHS Trust

Jose Bastos

Christopher Broomhead

Lorell Brownlee

Gregory Burch

Marta Campbell

Rory Carrigan

Hadia Farooq

Danielle Fawkes

Geetha Gunaratnam

Lucy Guthrie

Naomi Hancox

Natasha Kennedy

Mehnaz Khan

Andy Kwok

Hannah Leaman

Queenie Lo

Nurhayati Lubis*

Katherine MacGloin

Vijeta Mahinthan

Otto Mohr

Angela Nicklin

Olubukunola Ojo

Constantinos Papoutsos

Katherine Railton

Amrit Roopra

Simon Stacey

Basildon and Thurrock University Hospitals NHS Foundation Trust

Samuel Armanious*

Karen Culfear

Bedford Hospital NHS Trust

Douglas Bomford

Anand Desai

Preeti Dewan

Henry Hammerbeck

Michael Holland

Peter Knowlden*

Ben Linton Willoughby

Andrew Mckendry

Muhammad Nasiruddin

Alessandra Reis-Clark

Helena Stafford

Birmingham Women's NHS Foundation Trust

Khalid Hasan

Yasmin Poonawala

Annabelle Whapples*^i^

Blackpool Teaching Hospitals NHS Foundation Trust

Emma Brennan

Janette Brown

Rosanne Ching

Jason Cupitt*

Karen Gratrix

Katherine Hodgson

Sean O'Beirn

Dennis Padi

Christopher Pemberton

Maja Severn

Bolton NHS Foundation Trust

Jon Arnot-Smith

Hugo Buckley

Samuel Howell^c^

Ruth Loeffler^c^

Susan Moss

Seelanere Nandini

Peter Sandbach*^c^

Emma Wheatley

Bradford Teaching Hospitals NHS Foundation Trust

Richard Briscoe

Benjamin Brown

Sarah Campion

Sarah Cooper

David Craske

Peter Hart

Tom Lawton

Maria Marta ^a^

Taha Namik

Adam Neep

Martin Northey

Rashmi Senaratne

Robert Spencer*

Helen Stanworth

Mark Stubbington ^a^

Amit Surah ^a^

Brighton and Sussex University Hospitals NHS Trust

James Jack

Jessica Lee

Toni Perello Sancho

Stuart White*

Buckinghamshire Healthcare NHS Trust

Claire Ansley-Watson

Alice Pearl Christensen

Jonathan Collins

Jeremy Drake*

Phil Duggleby

Helen Gilfillan

Samantha Jayaweera

Mariam Latif

Alice Loft

Tamsin McAllister

Jyoti Misra

Amanda Mohabir

Vanisha Parekh

Hasita Patel

James Robert Sheehan

William John Stevens

Nicholas Taylor

Ravi Tomar

Emily Traer

Elizabeth Yates

Burton Hospitals NHS Foundation Trust

Seetal Aggarwal

Catriona Frankling

Georgia Knight ^i^

Clare Mewies

Eleanor Tyagi*

Carole Webb

Calderdale and Huddersfield NHS Foundation Trust

Sarah Elizabeth Hennell

Stephen John Hill*

Amy Mayor

David Pegg

Amanda Pine

Sally Price

Amit Pruthi

Cambridge University Hospitals NHS Foundation Trust

Famila Alagarsamy

Jim Bamber*

Jo Bytham

Ailsa Liddle

Bassey Brendan Nkanang

Georgina Singleton

Helen Surgenor

Jacqueline Tahari

Kelly Teversham

Shanelle Wijesuriya

Central Manchester and Manchester Children's University Hospitals NHS Foundation Trust

Dilip Agarwal^c^

Hassan Ahmad^c^

Niamat Aldamluji^c^

Sujesh Bansal*^c^

Neeraj Bhardwaj

Kailash Bhatia*^c^

Tara Byott^c^

Daniel Conway

Devjay Datta

Charlotte Dean

Nick Greenwood*^c^

Amr Hassan^c^

Mohammad Ibrahim

Matthew James Jackson^c^

Amit Jain

Maciej Kalata

Swati Karmarkar*^c^

Helen Lewis^c^

Emma Lightbody

Mohamed Mahmoud

Ijas Moideen

Niall O'Keeffe

Chinmay Patvardhan^c^

Anand Rajasekaran^c^

Richard Ramsaran^c^

Isma Razzaq

William Simpson^c^

Rebecca Sutton*^c^

Mruthunjaya Swamy*^c^

Katalin Szabo

Cheng Yeow Tan^c^

Chelsea and Westminster Hospital NHS Foundation Trust

Laura Braidford

Rhian Bull

Jaime Carungcong

Ivy Chan

Alice Costello

Ruth Cowen

Mark Cox

Kevin Haire

Katherine Horner

Inga Kelpanides

Ami Kotecha

Kylie Norrie

Tejal Patel

Amanpreet Sarna

Sneh Shah

Valeria Silvestre

Patrick Ward

Andrea Weigert*

Michael Weston

Harriet Wordsworth

Chesterfield Royal Hospital NHS Foundation Trust

Sarah Capper ^d^

Sian Edwards

Rachel Gascoyne

Sue Glenn

Emily Hall

Elaine Harris

Mohammed Khan

Matthew Needham ^d^

Anand Padmakumar

Nick Spittle* ^d^

Lesley Stevenson

Julie Toms

Caroline Wearn

Amanda Whileman

Janina Woodward

Christie Hospital NHS Foundation Trust

Nesrine Ismailn

Jaya Nariani*

Rafik Sedra

City Hospitals Sunderland NHS Foundation Trust

Lynn Fairless

Dawn Keightley

Eleanor Ripley

Marc Slorach*

Colchester Hospital University NHS Foundation Trust

Richard Bayley

Debbie Campbell

Alison Ghosh

Samuel Kestner

Ram Kumar

Rachel Madders

Thomas McLoughlin*

Sophia Paramanathan

Peter Wicks

Countess of Chester Hospital NHS Foundation Trust

Mary Aldous

Jonathan Breeze

David Castillo*

Jenny Davies

Angela Deeley

Tom Donaldson

Michaela Duskova

Jamie Fanning

Maria Faulkner

Alain Fennessy

Brian Herath

Mark Howells

Helen Jeffrey

Anita Jhamatt

Gregory Moore

Ben Murray

Helen Robertson

Nicole Robin

Robert Neil Schofield

Eoin Young

County Durham and Darlington NHS Foundation Trust

Chloe Barclay

Sabrina Butcher

Helen Chipchase

Amanda Cowton

Lynsey Downing

Monica Jackson^b^

Anthony Kennedy

James Limb*

Mihaela Michituc

Kannan Mohanram

Mandy Porritt

Katherine Rouse

Priya Shekar

Alison Thompson

Linda Tinkler

Croydon Health Services NHS Trust

Srinivasan Dhileepan*

Richard Doyle

Irfan Raza

Holly Sturgess

Derby Hospitals NHS Foundation Trust

Marie Appleby

Sallyann Bell

Trish Boateng

Teresa Grieve

Kathleen Holding

Ryan Humphries

Melody MacGregor

Susan Melbourne

Yvonne Newey

Karen Pearson

Qiong Pooler

Manni Sandhu

Karen Simmonds

Coral Smith

Jill Smith

Shafa Ullah

Vanessa Unsworth

John Williams*

Doncaster and Bassetlaw Hospitals NHS Foundation Trust

Sunita Asif ^d^

Mary Avvai

Amira Bassaly

Paul Bedford

Maneka Braganza ^d^

Michelle Denton

Richard Dobson* ^d^

Agota Ermenyi

Ashraf Fakhry Farid

Mark Fowler

Padma Gopal

Aditya Krishan Kapoor ^d^

Weeraman Karunaratne

Rachel Lee

Shanmugam Narayanaswamy

Matthew Neal

David Northwood ^d^

Christopher Palmer

Joel Perfitt ^d^

Javaid Rashid

Najam Saqib

Parul Talati

Tim Vemmer

Vinesh Vincent B

Ben Williams

Dorset County Hospital NHS Foundation Trust

Jonathan Chambers*

Russell Goodall

Erica Jolly

Stephanie Jones

James Keegan

Sarah Moreton

Jai Vedwan

Dudley Group NHS Foundation Trust

Michael Allan

Clare Allcock

Roisin Baker ^i^

Felicity Corcoran

Phillip Howells

James Hutchinson

Sarah McCormick

Ciro Morgese

Richard Pierson* ^i^

Julian Sonksen

Rebecca Storey

Merotra Susan

Ben Watkins

Ealing Hospital NHS Trust

Leena Ali*

Shahrzad Pakkar Tadbiri

Seosoon Seah*

East and North Hertfordshire NHS Trust

Siân Batley

Shanmugasundaram Gowrie-Mohan*

Yohinee Rajendran

East Cheshire NHS Trust

Kieran Bowdren

Andrew Gorman*

East Kent Hospitals University NHS Foundation Trust

Sanjay Agrawal

Farrukh Ameer

Roya Bayliss

James Bland

Claire Braid

Rajkumar Johi*

John MacKinnon

Joanna Moore*

Nagendra Natarajan*

Rini Poddar

Rebekah Rodgers

Rebecca Samuels

Andrew Skinner

Christopher Turner

East Lancashire Hospitals NHS Trust

Marion Anderson^c^

Kate Beresford*

Srikanth Chukkambotla

Hawa Desai

Janie Hartley

Siobhane Holden^c^

Farzana Ibrahim

Matthew Leliuga

Duncan MacRosson^c^

Aji Mathew*^c^

Michael Pollard

David Rawlinson

Haroon Waqar-Uddin

John Weeks

East Sussex Healthcare NHS Trust

Anthony Cochrane

Leon Dryden

Venkatesan Duraiswamy

Philippa Marshall*

Amanda Milligan

Roisin Monteiro

Seema Pai

Frantisek Slaninka

Claire Stikans

Epsom and St Helier University Hospitals NHS Trust

Andrew Bailey

Caroline Bullen

Joan Desborough

Tasneem Katawala

David Male

Amrut Mudanna

Jasmina Perinpanayagam

Franca Serafini

Geoff Thorning*

Frimley Park Hospital NHS Foundation Trust

Victoria Atkinson

Claire Cameron

Merle Cohen

Britt Garwood

George Godfrey

Sara Lantz-Dretnik

Ana Robles

Justin Woods*

Gateshead Health NHS Foundation Trust

Connor Dooley

Vanessa Linnett*

Stephen Mowat^b^

Jenny Ritzema

Louise Sanderson

George Eliot Hospital NHS Trust

Divya Khare

Vivek Poongavanam* ^i^

Manoj Ravindran

Gloucestershire Hospitals NHS Foundation Trust

Katherine Belfield

Emily Buckwell

Stephanie Connelly

Amy Dodd

Paul Downie*

Charlotte Earnshaw

Natalie Gray^f^

Sarah Major

Kelly Matthews

Victoria Ormerod

Nishi Patel

Sophie Scutt^f^

Kathleen Shelley

Laura Thomson^f^

Carolyn Warr*

Great Western Hospitals NHS Foundation Trust

Sam Andrews

Gary Baigel*

Johanna Mockler

Jon Rivers

Lucy Williams

Guy's and St Thomas' NHS Foundation Trust

James Arlidge

Victoria Bennett

Alexandra Bond

Arun Kochhar

Chandni Parikh

Jan Schumacher*

Agnieszka Skorko

Davina Wong

Hampshire Hospitals NHS Foundation Trust

Samantha Allen

Helen Bromhead*

Alison Corker

Victoria Corner

Angie Dempster

Olatunde Fagbayimu

Johanna Hellstrom

Jane Martin

Lewis Matthews

Philip McGlone ^e^

Richard Partridge*

Emma Rogers

Angus Royal

Joanne Tambellini

Dawn Trodd

Nimu Varsani

Caroline Wrey Brown

Louise Young ^e^

Harrogate and District NHS Foundation Trust

Jennifer Firth-Gieben

Sarah Peacock

Abhinav Kant*

Chris Smales

Heart of England NHS Foundation Trust

John Benjamin

Gemma Dignam ^i^

Hannah Fuller

Saravanababu Gnanaseakaran*

Bethan Hale ^i^

Vivienne Madden

Alan Marshall

Sandur Naresh

Priya Ramchandran

Sethu Veerabadran*

Vijay Kumar Venkatesh

Guenter Weissenhorn

Hillingdon Hospitals NHS Foundation Trust

Charlotte Stephanie Cattlin

Francesca Mazzola

Constandinos Papageorgiou*

Shivali Patel

Hinchingbrooke Health Care NHS Trust

Aisling Connolly

Saad Gomaa

Dancho Ignatov*

Zhana Ignatova

Ilya Kantsedikas

Namratha Mathai

Mohammad Tayyab Memon

Ben Miller

James O'Brien

Georgina Singleton

Arivalagan Sivakkolunthu

Sivaprakash Vaitheeswaran

Homerton University Hospital NHS Foundation Trust

Emily Bayne

Jens Full

Hoon Ying Lau

Winston Ng

Sandor Orosz

Tabitha Tanqueray*

Andrew Wilkinson

Hull and East Yorkshire Hospitals NHS Trust

Caroline Abernethy

Rachel Bowden ^a^

Pallavbhai Desai ^a^

Omer Farooq ^a^

Andrew Gratrix*

Anne-Mair Hammond-Jones ^a^

Vikas Kaura ^a^

Umakanth R Kempanna ^a^

Victoria Martinson

Muhammad Shakeel Riaz ^a^

Amjad Sami

Neil Smith

James Stevenson ^a^

Ruth Ugochukwu

Mamoon Yusaf ^a^

Imperial College Healthcare NHS Trust

Melloney Allnutt

Glenn Arnold*

Satya Bhat*

Nicola Boyer

Rachel Freedman

David Harris

Gillian Hornzee

Asantha Jayaweera

Craig Jones

Ashwin Kalbag

Shamsher Khan

Kiranjit Khazan Singh

Zheyna Konstantinova

Stuart Lawrie

David Magee

Nisha Nimalan

Laura Peltola

Rajan Saini

Marco Scaramuzzi

Akshay Shah

Ipswich Hospital NHS Trust

Alice Barnes

Debra Beeby

Stephanie Bell

Lyndsey Cubitt

Daniel Gibney

Louise Hunt

Arun Natarajan*

Margaret Oatley

Genessa Peters

Bally Purewal

Judi Ramsey

Joanne Rickett

Ginny Rose

Stephanie Stoddart

Edward Watson

Isle of Wight NHS Trust

Gabor Debreceni*

Laszlo Ordog

Isobel Rice

Souvik Sanyal

James Paget University Hospitals NHS Foundation Trust

Dushani Atapattu

Pieter Bothma*

Kiran Dasari

Tammy George

Abby Greaves

Christian Hacon

Pushpalatha Linga Nathan

Kettering General Hospital NHS Foundation Trust

Duncan Baines

Anjali Bilolikar

Shiv Chande

Satyanarayana Jakkampudi*

Jeannie Kessell

Prakash Krishnan

Pawan Kumar

Jo Novaga

Parizade Raymode

Philip Watt

Zoe Whitman

Elinor Wighton

King's College Hospital NHS Foundation Trust

Daniel Abell

Michael Adlam

Luke Banks

Nadia Blunt

Philip Hopkins*

James Gill

Roshmi Kumar*

Alena Mulenga Skupenova

George Perrett

Andrew Selman

Jasjot Kaur Singhota

Helen Statham*

Kingston Hospital NHS Foundation Trust

Tharumalingam Gowripalann

Rumiko King

Sarang Puranik*

Lancashire Teaching Hospitals NHS Foundation Trust

Mark Children

Ian Clegg

Karen Collins

James Garbarino^c^

Andrew Hall

Maurice John Jones*^c^

Jessica Maycock

Andy Ng

Jennifer Pettit^c^

Alison Quinn^c^

Grace Singleton

Yenyeen Teh^c^

Thomas Walton

Tom Whitehead

Amanda Zacharzewski

Leeds Teaching Hospitals NHS Trust

Ossian Auckland-Child ^a^

Zoe Beardow

Christopher Bull ^a^

Anna Costello ^a^

Stuart Elliot

Michal Luniewski ^a^

Beverley Parker

Papanna Ramakrishnan

Caroline Thomas ^a^

Matthew Tinker

Brian White*

Simon Whiteley*

Lewisham and Greenwich NHS Trust

Manju Agarwal*

Seb Baxter

John Cronin

Liverpool Heart and Chest Hospital NHS Foundation Trust

Seema Agarwal*

Peter Delve

Sianedd Elliott

Gerald Smith

Liverpool Women's NHS Foundation Trust

Philip Barclay*

Emily Christie

Luton and Dunstable Hospital NHS Trust

Suhanya Allirajah

Edward Dewhirst

Benjamin Marriage

Natalia Notkina

Imran Razzaq

Emily Spence

Suhail Zaidi*

Maidstone and Tunbridge Wells NHS Trust

Philip John Blackie*

Dawn Harpham

Catherine Lloyd

Tracey Nolan

Charles Prior

Medway NHS Foundation Trust

Jon Aron

Bryony Davy

Kirtida Mukherjee*

Brenda Nyamaizi

Mid Cheshire Hospitals NHS Foundation Trust

Zaherali Damani

Penelope Edmondson

Fozia Hayat

Nicolas Price*

Laura Wilson

Mid Yorkshire Hospitals NHS Trust

Philip Antill

Adam Beaini

Matthew Bromley

Rob Charles ^a^

Robert Jackson ^a^

Rebecca Lathey ^a^

Mohamed Lklouk

Oliver Martin

Irfan Mohammad Omar

Alastair Rose

Oliver Ross-Smith ^a^

Anupam Sharma

Karen Simeson

Annika Smith

Rachel Stock

Simon Timperley ^a^

Sandeep Varma*

Milton Keynes Hospital NHS Foundation Trust

Ramesh Achari

Chrissie Chevis

Radu Chirvasuta

Veronica Edgell

John Ekpa

Zahra Fazel

Manu Gupta

Nathan Huneke

Surekha Jani

Helen Jeffers

Amitabh Lahkar

Graziana Massolini*

Hayley Moss

Cheryl Padilla-Harris

Cherish Paul

Bernadetta Sawarzynska-Ryszka

Jeannette Smith

Moorfields Eye Hospital NHS Foundation Trust

Nicoletta Catteruccia*

Mhairi Jhugursing

Mohammed Kadim

Jonathan Mathers

Rutesh Mehta

Radha Sabaratnam

Newcastle upon Tyne Hospitals NHS Foundation Trust

Shylesh Aravindan

Emily Bonner

Thomas Cairns^b^

Mark Callaghan^b^

Nicholas Crawford-Upton

Christopher Gibb^b^

Jane Gibson

Ben Goodman^b^

Tom Haigh

Amy Holley

Linda McGhee^b^

Claire Mitchell^b^

Andrew Pollard

Thomas Edward Poulton

Sneha Prasad^b^

Edward Pugh^b^

David Saunders*

Sharon Scully*

Mirjana Starinac-Baldwin

Clare Watkinson^b^

Norfolk and Norwich University Hospitals NHS Foundation Trust

Siddharth Adyanthaya

Katrina Barber

Jian Wen Chan

Caron Dames

Amy-Jo Farrow

Jim Flatt

Grainne Garvey

Jane Hermanowski

Michael Irvine

Caroline Reavley*

Santhosh Kumar Thandayuthapani

Joanna Wheble

North Bristol NHS Trust

Alia Darweish^f^

Sarah Hoskins^f^

Kathryn Jenkins*

Sarah Martindale*

Katherine Jane Nickell

Gemma Nickols^f^

Biju Peringathara^f^

Sarah Todhunter^f^

North Cumbria University Hospitals NHS Trust

Hannah Crowther

Suresh Eapen

Gail Fitzsimmons*

Fiona Graham*

Leon Jonker

Nicci Kelsall

Savin Pokhrel

Una Poultney

Tracey Riley

Sarah Thornthwaite

Katie Williams

Toni Wilson

North Middlesex University Hospital NHS Trust

J. A. Ezihe-Ejiofor*

Hemantha Handapangoda

Rajesh Krishnan

Bonnie Kyle

Stefan Sevastru

North Tees and Hartlepool NHS Foundation Trust

Adrian Gooding

Mika Hamilton

Lisa Molus

Pradeep Orakkan

David Pritchard*

North West London Hospitals NHS Trust

Michael Berry

Jacek Borkowski

Thomas Fitzgerald*

Nicola Pearson

Ivan Wong

Northampton General Hospital NHS Trust

Swarna Athukorala

Sunjay Bhadresha

Rosemarie Carrick

Omur Choudhury

Prashant Kakodkar*

Andy Kempa

Senthil Kumaran

Akeeban Maheswaran

Ihaab Matabdin

Saurabh Mehrotra

Kate Smith

Jennifer Spimpolo

Ulrika Winstone

Northern Devon Healthcare NHS Trust

Geraldine Belcher

Garry Henry*

Nigel Hollister

Rachel Horn

Jane Hunt

Maria Karatzi

Eleanore Quinn^g^

David Robertshaw

Amanda Skinner

Lucia Stancombe^g^

Northern Lincolnshire and Goole Hospitals NHS Foundation Trust

Abbey Clark ^a^

Sanjeev Garg*

Priyadarshan Potla

Andrew Quin ^a^

Anju Raina

Roshan Rao ^a^

Mustafa Raza

Northumbria Healthcare NHS Foundation Trust

Maureen Armstrong

Mark Dalton

Joanne Dawson

Leonie English

Neil Owen Hall

Renee Hope

Thomas Mackie

Fiona McMenemie

Joseph Nevin

Adrian Taylor*

Nottingham University Hospitals NHS Trust

Michal Czernicki

Corinne Deakin

Simon Denning

Prerna Mehrotra

Iain Moppett*

Thomas Munford

Matthew O’Meara

Katie Samuel

Deborshi Sinha

Ganna Sliusar

Deepak Subramani

Oxford University Hospitals NHS Trust

Vassilis Athanassoglou

Amisha Burumdayal

Kim Carter

Alexandra Crean

Ian Edmond

Soumitra Ghosh

Cyndi Goh

Eleanor Harvey

Luis Lee

Nicholas Love

Laila Morris

Georgina Neall

Amy Sangam

Shaun Scott*

Papworth Hospital NHS Foundation Trust

Joanne Irons

Rebecca McClean

Amy Needham

Elizabeth Ogilvie

Kiran Salaunkey

Stephen Webb*

Pennine Acute NHS Trust

Salmin Aseri^c^

Sarah Babatunde^c^

Amit Bhargava^c^

Juie Cunningham

Boopathy Dhanasekar*^c^

Alastair Duncan^c^

Jennifer Gwinnutt^c^

Holly Hammond^c^

Joanne Humphreys^c^

Linda Kent

Owen King^c^

Benjamin Kolb^c^

Julie Kuzhively^c^

Juliette Li Wan Po^c^

Denise McSorland

Zahaan Wayne Pinto

Bhaskar Saha*^c^

Jo Taylor

Paula Usher

Ming Wilson^c^

Peterborough and Stamford Hospitals NHS Foundation Trust

Najwan Abu Al-Saad

Balraj Appadu*

Karen Beaumont

Christopher Hall

Nazia Khan

Rachelle MacMillan

Plymouth Hospitals NHS Trust

Rohan Babla^g^

Charlotte Bailey

Molly Bancroft

Karen Brookes

Leeanne Carwithen

Helen Davies

Juleen Fasham^g^

Danielle Franklin^g^

Julie Greatrex

Rosie Griver^g^

Catherine Harden

Craig Holdstock^g^

Matt Hawkins

Sandeep Kusre^g^

Val Morgan

Gary Minto*^g^

Claire Northcott

Abigail Patrick

Suzanne Price

Jeanne Louise Schonborn^g^

Ellie Shepherd

Rosalyn Squire

Geoff Tavener^g^

Liz Whitby

Jackie Wooding

Poole Hospital NHS Foundation Trust

Katharine Browne

Hermione Denniss

Tom Everett

Michael Girgis*

Harris Wain

Portsmouth Hospitals NHS Trust

James Bain*

Adam Edwards

Renee Ford

Rebecca Fry

Li Ping Gan

Helen Gordon

Sophia Henderson ^e^

Susannah Palmer

Rebecca Reeves

Francesca Riccio

Esme Sleap

Zoe Smith

Queen Victoria Hospital NHS Foundation Trust

Julian Giles*

Valentina Neroli

Isabelle Reed

Debbie Weller

Robert Jones and Agnes Hunt Orthopaedic Hospital NHS Foundation Trust

Rob Alcock

Robert Banerjee

Andrew Bing

Sumeeta Conry

Jayne Davies

Kirsty Davies

David Ford

Robert Freeman

Peter Gallacher

Niall Graham

Stuart Hay

Simon Hill

David Jaffray

John John*

Huw Jones

Fouzia Kader

Siddalingaprabhu Katti

Julia Kennedy

Nigel Kiely

Ruth Longfellow

Ciaran Moran

James Neil

Matthew Ockendon

James Pattison

James Richardson

Richard Roach

Sophie Shapter

Richard Spencer-Jones

Niall Steele

Julie Steen

Jayesh Trivedi

Marck Vanliefland

Emma Wain

Huw Williams

Rotherham NHS Foundation Trust

William Lindsay ^d^

Frances Colquhoun ^d^

Matthew Craig Faulds ^d^

Meredyth Harris

Anil Hormis* ^d^

Joanne Howe

Sean Keating ^d^

Amy Thomas ^d^

Royal Berkshire NHS Foundation Trust

Tamara Alexander

Richard Barnes

Jennifer Cade

Joanna Cudlipp

Patrick Dill-Russell*

Henry Jefferson

John MacKenzie

Gopalan Radhakrishnan

Royal Bournemouth & Christchurch Hospitals NHS Foundation Trust

Duncan Chambler

James Craig*

Emily Cross

Stephen Phillips

Paul Stevens

Emma Willett

Steven Williams

Laura Wood

Royal Brompton and Harefield NHS Foundation Trust

Rob Anker

Tali Heymann

Katheryn Fogg*

Sandra Reinero

Aikaterini Vlachou

Royal Cornwall Hospitals NHS Trust

Alyson Andrew

Weiss Anna

Amanda Datson

Nicki Devooght-Johnson

Jacqueline Dingle

William English

Anna Fouracres

Juan Graterol*^g^

Lynne Graves

Alexander Ishimaru

Patrycja Jonetzko^g^

Keely Lane

Tom Lawson^g^

Andrew MacAlister Hall^g^

Kathleen Mulcahy^g^

Anna Ratcliffe^g^

William Rutherford^g^

Jessica Summers

Gina Townley

Lisa Trembath

Leanne Welch

Geoffrey Wigmore^g^

Royal Devon and Exeter NHS Foundation Trust

Elizabeth Fontaine^g^

Emily Howells^g^

David Hutchins^g^

Lawrence Kidd^g^

Samantha Lyons^g^

Katharine Meikle*^g^

Richard David Wassall^g^

Thomas Woodward^g^

Royal Free London NHS Foundation Trust

Ciara Donohue

Faezeh Godazgar

Charlotte Haldane

Sarah James

Carlos Kidel

Sue Mallett*

Edward Palmer

Royal Liverpool and Broadgreen University Hospitals NHS Trust

Jonas Appiah-Ankam

Jane Beattie

Jennifer Burgess

Laura Dagg

Nirmal Daniel*

Joanna Fawkner-Corbett

Will Gauntlett

Sally Hargreaves

Michael McGovern

Sarah Mitchell

Christopher Parker

Sergey Rastopyrov

Alicia Roitberg-Henry

Maria Safar

Royal Marsden NHS Foundation Trust

Oliver Blightman

Stuart Cleland

Shaman Jhanji*

Rohit Juneja*

Helen Jane Lawrence

Sophie Uren

Royal National Orthopaedic Hospital NHS Trust

Anamika Agrawal

Paul Gunning*

Henna Khetani

Benjamin Parsons

Royal Orthopaedic Hospital NHS Foundation Trust

Chandra Bhimarasetty

Laura Bird

Dinakar Gowda

Rekha Jayapal

Faye Moore

Salmaan Mughal

Sam Papadopoullos

Narendra Babu Siddaiah* ^i^

Royal Surrey County Hospital NHS Foundation Trust

Sophie Childs

Matthew Dickinson*

Andrew McKechnie

Frederick Wilson

Royal United Hospital Bath NHS Trust

Cheryl Achary^f^

Jeremy Astin^f^

Nicholas Harris

Lesley Jordan*

Inthu Kangesan

Catherine Lomasney

Melanie Mcdonald

Royal Wolverhampton Hospitals NHS Trust

Rebecca Coles

Kate Griffin

Richard Lightfoot

Rebecca Micklewright*

Linga Prasad ^i^

Sreekanth Uppugonduri*

Salford Royal NHS Foundation Trust

Charlotte Ash

Cally Burnand*^c^

Richard Cooper

Simon Forrington

Hywel Garrard

Alison Jones^c^

Leigh Willoughby*^c^

Salisbury NHS Foundation Trust

Alpha Anthony

Caroline Clarke

Ilana Delroy-Buelles ^e^

Christian Schopflin ^e^

Andrew Swain

Simon Williams*

Sandwell and West Birmingham Hospitals NHS Trust

Mary Cheung

Kavit Dasari

Rachael Dolan

Phillip El-Dalil

Katheryn Grange

Gunasheela Kalashetty

Santhana Kannan* ^i^

Arif Jamil Khan

Laura Kocierz

Frances Lay

Miriam Namih ^i^

Harry Phillips

Mrutyunjaya Rao Rambhatla*

Tracy Wong

Sheffield Teaching Hospitals NHS Foundation Trust

Hemant Aaytee ^d^

Norfaizan Ahmad ^d^

Sireesha Aluri ^d^

Kris Bauchmuller ^d^

Helen Ellis ^d^

Zakir Hajat ^d^

Holly Jeffery ^d^

Alex Kojro ^d^

Andrew Cruikshanks ^d^

Andrew Leeson ^d^

Duncan Miller ^d^

Rama Pothireddy ^d^

Ajay Raithatha ^d^

Catherine Riley ^d^

Jonathan Rosser ^d^

Rachel Louise Wadsworth ^d^

Ian Wrench* ^d^

South Devon Healthcare NHS Foundation Trust

Martin Dore^g^

Lydia Jones^g^

Chris Leighton^g^

Jane Montgomery*^g^

Claire Ward^g^

South Tees Hospitals NHS Foundation Trust

Rasha Abouelmagd

Lukasz Badek

Kirsty Baillie

Anita Baldea

David Booth*

Louise Cawthorn

Emanuel Cirstea

Kerry Colling

Lauren Crook

Anand Damodaran

Mohamed Eid^b^

Janet Gunn

Christoph Muench*

Anand Nadgir

Rebecca Parker

Kavita Upadhyaya

South Tyneside NHS Foundation Trust

Gayle Clifford

Christian Frey*

Shiv Gurung

Philippa Laverick

South Warwickshire NHS Foundation Trust

Alastair Fairfield

Emert White*

Southend University Hospital NHS Foundation Trust

Kapil Arora

Blanca Boira*

Simon Matthews

Karen Rhodes

Maylan Webb

Southport and Ormskirk Hospital NHS Trust

Andrew Kent*

Helen Terrett

St George's Healthcare NHS Trust

Roopa Sindhu Devanahalli*

Victoria Eleanor Crozier Ferrier

James Hayward

Clare Ivermee

Anastasia-Eleni Legga

Laura Litster

Georgia Monantera

Ben Morrison

Rita Saha

Guy Sanders

Natashia Schneider

Nikunj Shah

Rohan Vandabona

James Wilson

St Helens and Knowsley Teaching Hospitals NHS Trust

Andrew Fisher

Vandana Goel

Preeti Kuduvalli*

Dominic Peter Douglas Nielsen

Kim Porter

Stockport NHS Foundation Trust

Janette Curtis

Sophie Jagatia

Deborah Lee

Leila Nasser

Sarah Scanlon

Helen Simmons^c^

Elizabeth Thomas*^c^

Surrey and Sussex Healthcare NHS Trust

Olivia Davies

Hannah Dawe

Gannat Gabr

Ashley Hague

Richard Hawkins

Christie Locke

Aikaterini Papadopoulou

Simon Parrington*

Vijaya Ramaiah

Anna Riccoboni

Elizabeth Smee

Frederick Van Damme

Matthew Willcocks

Tameside Hospital NHS Foundation Trust

Anand Kulkarni*

Venkata K Melachuri*^c^

John Nicholls

Hannah Richardson^c^

Laura Stephanie Talbot^c^

Stephen Wythe^c^

Taunton and Somerset NHS Foundation Trust

Lindsey Arrick^g^

Stuart Collins*^g^

Thomas Evans^g^

Harriet Gardiner^g^

James Garwood^g^

Olivia Gokhale^g^

Suzanne Grenfell^g^

Alexander Jones^g^

Rebecca Pugsley^g^

Nicholas Soulter^g^

Borys Topor^g^

Tim Wilson^g^

The Princess Alexandra Hospital NHS Trust

Natasha Constandinou

Joanna Hackney

Kevin Hamilton*

Jenna Hutchinson

Michelle Reichman

The Whittington Hospital NHS Trust

Karen Fan

Heloise Hayakawa

Jane Silk*

Duncan Wagstaff

United Lincolnshire Hospitals NHS Trust

Simon Archer

Helen Ayre

Susie Butler

Matthew Daunt

Val Elliott

Joanne Fletcher

Sarah Ford

Yusuf Ghumra

Anne Hardwick

Suganthi Joachim*

Manish Kakkar*

Amy Kirkby

Jonathan Lewis

Ann Marsh

Beverley Mashegede

Madhur Mehta

Kimberley Netherton

Rebecca Norton

Tara Palmer

Natalie Reinoso

Amanda Roper

Victoria Sherburn

Andrew Sloan

Isobel Thomas

Maria Tute

University College London Hospitals NHS Foundation Trust

Douglas Blackwood

Sara Bowman

Maria Chazapis

Roger Cordery*

Tejal Dave

Catriona Ferguson*

Roxaan Jappie

Sathish Kumar

Mark Lambert

Leda Lignos

Valpuri Luoma*

Emilie Martinoni Hoogenboom

Joanna Mavridou

Vidhya Nagaratnam*

James Pennington

Upeka Ranasinghe

Ramai Santhirapala

Sohini Sengupta

Louisa Shovel

Serena Sodha

Adrienne Stewart*

Julia Taylor

Manni Waraich

University Hospital of North Staffordshire NHS Trust

Beryl Alcock

Felicity Clark

Viv Colclough

Sarah Dawson

Jane Delaney

Satyajeet Ghatge

Ellen Haire

Anne Harrison

Ellen Hodgson

Rahul Kumar*

Elin Jones

Piotr Ohly

Nicola Pattison

Marissa Plaza

Mohan Sathyamoorthy

Lisa Wilkings

University Hospital of South Manchester NHS Foundation Trust

Sarah Blomeley^c^

Jennifer Cunningham

Rebecca Elder

Johnny Kenth

Matthew Leech^c^

Wendy Nichols

Alison Walker

Stephen Washington*^c^

Brian Williams^c^

University Hospital Southampton NHS Foundation Trust

Samar Al-Rawi*

Yousra Ahmad

Clare Bolger

Anne-Marie Bougeard

Hannah Collins

Rhys Davies

Kim Golder

Alexandra Hawken

Lesley Hawkins

Thomas Hutley

Alexa King

Denny Levett

Angela Lim

Nihal Mahagamage

James Montague

Andrew Nash

James Plumb

Karen Salmon

Leanne Seaward

Anamika Sehgal

Laura Tompsett

Bryony Tyrell

Andrew Vaughton

Beverley Wadams

Mai Wakatsuki*

University Hospitals Birmingham NHS Foundation Trust

Muneeba Ahmed ^i^

Somasundaram Jeyanthan

Katherine Laver ^i^

Rachel Moore

Hasnain Saeed

Laura Tasker*

University Hospitals Bristol NHS Foundation Trust

Andrew Bartlett

Hannah Blanshard

Alan Cohen

Helen Davies^f^

Nirosha De Zoysa

Shailendra Deep

Claire Dowse

Chris Gough

Lisa Grimmer

Ben Howes

Sarah Jarvis

Attila Jonas

Neil Muchatuta

Yin Ng^f^

Marcin Pachucki^f^

Claudia Paoloni*

Helen Parker

Tamara Semei-Spencer

Nicola Stewart

Sarah Thomas

Hannah Wilson

Nicholas Wharton*

University Hospitals Coventry and Warwickshire NHS Trust

Carol Bradbury*

Falguni Choksey

Nageena Hussain

Ahmed Mesbah

Bradley Mullins

Arif Qureshi

Chandra Vaidyanath

University Hospitals of Leicester NHS Trust

Matthew Charlton

Navreet Ghuman

Sameer Hanna-Jumma

Stephen Hillier

Jim Horn

Kaushliya Katechia

Gary Lau

Ralph Leighton*

Jennifer Norris

Sarah Bowrey

University Hospitals of Morecambe Bay NHS Foundation Trust

Claire Bartlett

Karen Burns

Kate Lloyd

Stephanie McGrath

Stephanie Monks

Matthew Dominic Newport

Anastasia Price

Corinne Rimmer*

Andrew Smith

Insiya Susnerwala

Clare Tibke

Walsall Healthcare NHS Trust

Chris Clulow

Gareth Lodwick

William Malein

Oliver Oxenham

Sumant Shanbhag*

Marc Whitehouse

Warrington & Halton Hospitals NHS Foundation Trust

Seema Charters*

Timothy Furniss

John Gardner

Chetana Kataria

Rachel Leopold

Sumit Mitra

Natasha Permall

Peter Turton

West Hertfordshire Hospitals NHS Trust

Malthi Dudley*

Aditi Ghei

Sumit Jha

Richard Lin

Ratna Makker

Jennifer Taylor

Elaine Walker

West Middlesex University Hospital NHS Trust

Edward Amiry

Jonathan Berger

Lie Wah Johnson

Alexandra Matson*

Tim Peters*

Metod Oblak

Cosmo Scurr

Lilia Tony

Thomas Young

West Suffolk NHS Foundation Trust

Joel Chin

Vijayakumar Gopal*

Lisa Grimes

Sally Humphreys

Laura Kessack

Katharina Kohler

Rajesh Vasiraju

Western Sussex Hospitals NHS Foundation Trust

Sonia Akrimi

Anna Cave

Charlotte Crossland

Paul Crowest

Emily Dana*

Rachael Grimaldi

Katherine Hunter

James Nicholson*

Zehra Ozfirat

Laura Frances Pocock

Michael Robson

Hannah Rose

William Shippam

Patrick Thorburn

Stuart Wade

Weston Area Health NHS Trust

Donna Cotterill

Alex Dewar

Guru Hosdurga*

Rik Marsden

Ed Mew^f^

Vivienne Pixton

Glenn Saunders

Wirral University Teaching Hospital NHS Foundation Trust

Sharon Acheson

Laura Bubb

Matthew Gwinnutt

Amy Hill

Neil Oakes

Suresh Singaravelu*

Worcestershire Acute Hospitals NHS Trust

Kate Blyth

Karen Faulkner

Emily Johnson ^i^

Ravindra Mallavalli

Christopher Mowatt* ^i^

Cindy Persad* ^i^

Emma Plunkett

Alag Raajkumar

Meghna Sharma

Naginder Singh

Wrightington, Wigan and Leigh NHS Foundation Trust

John Barrett

Simon Bluhm

Maria Brereton^c^

Paul Andrew Damon Clements*

Joanne Farnworth

Linzi Heaton

Diane Heaton

Julie Melville

Valerie Parkinson

Bryony Patrick

Jessica Sykes

Tracey Taylor

Wye Valley NHS Trust

Matthew Creed

Joseph Hayward

Richard Hodgson*

Aled Morgan

Yeovil District Hospital NHS Foundation Trust

Marmar Htyn^f^

Agnieszka Kubisz-Pudelko*

Ian Taylor^f^

York Teaching Hospital NHS Foundation Trust

Ramesh Ananthmanohar

Jessica Bowen

Lynne Brown

Andrew Chamberlain

Ben Chandler*

Kerry Deighton

Simon Dyer

Naomi Fleming ^a^

Muthuraj Kanakaraj*

Andrew McDonald ^a^

Eusebius U. Nworah

Altaf Hassan Sultanpori

Emma Temlett

James Walkington

Samantha Warnakulasuriya
